# Supplementary material for: Chromothripsis during telomere crisis is independent of NHEJ, and consistent with a replicative origin
Source: Genome Res. 2019 May;29(5):737–49. doi: 10.1101/gr.240705.118 (PMC6499312; doi:10.1101/gr.240705.118)
Supplement: Supplemental Material [file supp_gr.240705.118_Supplemental_file_1.zip › contigs/annotated_contigs/DB98/contig.2.DB98_length_681_mean_cov_8.37004405286.docx]

**DB98_length_681_mean_cov_8.37004405286**

```
ATCATTTATAAGATGTATGCGAAGATAGTTTGGGAATCTTATCTTGTTTAAGAACCAATATTATGAAAAATACCTATTATCTAATGGTC
 >chr2:167696219-167696497 - E=2e-155 p=4e-02
AAAAAGACAGATTGGATTAAATTTTATCTACCGACAATAGCTAAACAGTTTTTTAGGATTATTTCTTTATTTAAATAATATGGAATTCT

GAATTGAAGTTTCTGCCCCCATTTCATAGAAATGTGTAGTTTTTATTGTTTAGCTGATTCTGAAGCCACAACAATTGAACTAAGAGTTA

GTGCAATGC|AA|TAAATGGGACATATAGCTGTGGGAAAACAATGGATAATTTTATAAGATATAATAACTCCTCACACAACTCTTCATT
          >chr2:167695121-167695526 - E=6e-232
ACATTCATCAGAAAGAGAAACTATTAAAAATTTTAATATGGGGTCTATTAGACATTTCAGGACACATAAAACTTCTTTGAATTATGCTT

ATTTTTCAAAAATATCATATTAAATTTACTTGAGTTTTTAGGAAACAATATAATATGGACATTTTATATGTACAAACAAAAATATTTTT

TAAGTTACTTTGACATTTTGGATGTATGGCTGAATGAATAAATAATATATTTTACACTTGTCTTTGCTATATTTTTTAAAGCTGTATTA

ATTTTTAACTAGCCACTTTACTCAATCCTTTTTTGTTTTCATAATCAGGCAATTCTTTTG
```
